# The Response of the Estrogen-Related Receptor to 20-Hydroxyecdysone in *Bombyx mori*: Insight Into the Function of Estrogen-Related Receptor in Insect 20-Hydroxyecdysone Signaling Pathway

**DOI:** 10.3389/fphys.2021.785637

**Published:** 2022-01-18

**Authors:** Jinxin Wu, Guanwang Shen, Die Liu, Haoran Xu, Mengyao Jiao, Yungui Zhang, Ying Lin, Ping Zhao

**Affiliations:** ^1^Biological Science Research Center, Southwest University, Chongqing, China; ^2^State Key Laboratory of Silkworm Genome Biology, Southwest University, Chongqing, China; ^3^College of Sericulture, Textile and Biomass Sciences, Southwest University, Chongqing, China

**Keywords:** silkworm, estrogen-related receptor, 20-hydroxyecdysone, ecdysone response element, transcriptional activity, 20E signal pathway

## Abstract

Estrogen-related receptor (ERR) is an orphan nuclear receptor that was first discovered in animals, and play an important role in metabolism, development, and reproduction. Despite extensive research on the function of ERR, its transcriptional regulation mechanism remains unclear. In this study, we obtained the upstream region of *Bombyx mori ERR* (*BmERR*) and confirmed the promoter activity of this region. Interestingly, we found that 10 and 50 nM 20-hydroxyecdysone (20E) up-regulated the transcriptional activity of *BmERR* promoter. In addition, eight putative ecdysone response elements (EcREs) were predicted in the upstream sequence of *BmERR*. Based on their positions, the upstream sequence of *BmERR* was truncated into different fragments. Finally, an EcRE-like sequence (5′-AGTGCAGTAAACTGT-3′) was identified. Electrophoretic mobility shift assay (EMSA) and cell transfection experiments confirmed that this motif specifically binds to the complex formed between ecdysone receptor (BmEcR) and the ultraspiracle (BmUSP), a key complex in the 20E signaling pathway. Interference of *BmERR* or *BmEcR* mRNA in the embryonic cells of *Bombyx mori* significantly affected the expression of *BmEcR* and *BmUSP*. Overall, these results suggested that an EcRE element was identified from BmERR, and this will help understanding the detailed regulatory mechanism of ERR in insects.

## Introduction

Nuclear receptors are a large family of eukaryotic transcription factors (TFs) with important functions such as regulation of ligand-mediated gene expression and signaling pathways (Beato et al., 1995). Estrogen-related receptors (ERRs) are the orphan nuclear receptors and belong to the third nuclear receptor subfamily (NR3). Due to the high similarity in the ligand binding domain and DNA binding domain between ERR and estrogen receptor (ER), ERR can participating in the ER signaling pathway, sharing target genes, co-regulatory proteins, ligands and sites of action with the ER (Giguere, 2002; Greschik et al., 2002; Horard and Vanacker, 2003).

In mammals, there are three subtypes of ERRs, ERRα, ERRβ, and ERRγ, which play important roles in physiological and pathological functions (Deblois and Giguere, 2011). In particular, ERRs are closely associated with tumorigenesis. ERRα and ERRγ are two potential biological targets for the detection of breast cancer in women (Ariazi et al., 2002). ERRβ is shown to inhibit the growth of prostate cancer (Misawa and Inoue, 2015). In addition, ERRs play vital roles in energy metabolism, mitochondrial biogenesis, oxidative phosphorylation, fat metabolism, and cell growth (Carrier et al., 2004; Tremblay and Giguere, 2007; Deblois and Giguere, 2011; Eichner and Giguere, 2011; Sailland et al., 2014).

Most studies on ERR have focused on mammals. A homologous receptor of ERR has only been identified as a single subtype in insects. In *Drosophila*, ERR controls energy metabolism by regulating genes expression in the glycolytic pathway. ERR-knockout in *Drosophila* strains resulted in glycogen accumulation, and the glycolysis process was blocked, leading to death of the flies (Tennessen et al., 2011; Beebe et al., 2020). Genetic knockdown the expression of ERR in the testis of *Drosophila* inhibited the development of the testis and sperm production (Misra et al., 2017). ERR expression in *Apis cerana* and *Chironomus riparius* could be induced by external stress such as exposure to insecticides, ultraviolet rays, and fungicides (Park and Kwak, 2010; Zhang et al., 2016). Decreasing the expression ERR in male *Agrotis ipsilon* affected its sexual behavior (Bozzolan et al., 2017). To date, most studies on ERRs in insects focus on their physiological functions, whereas the mechanisms driving the regulation of ERRs remains unclear.

Since the completion of the genome sequence, the silkworm (*Bombyx mori*) has become a widely used model insect for lepidopteran research, and a general model organism for life sciences, toxicology, and fungal infections (International Silkworm Genome, 2008; Suetsugu et al., 2013; Meng et al., 2017; Abdelli et al., 2018; Nakajima et al., 2018; Matsumoto and Sekimizu, 2019). In previous research, we have demonstrated that the *Bombyx mori* ERR (BmERR) is involved in the growth and development of silkworm embryos and larvae by regulating the expression of glucose metabolism-related genes (Long et al., 2020; Shen et al., 2021). and vitellogenin *via* the 20-hydroxyecdysone (20E) -EcR pathway in silkworm (Shen et al., 2018). Therefore, we hypothesized that the activation of the 20E signaling pathway may be a key event in the transcriptional regulatory of ERR. To evaluate this possibility, we cloned the upstream sequence of *BmERR* including the transcription start site (TSS) using 5′-rapid amplification of cDNA ends (5′RACE), analyzed its transcriptional activity and relationship with 20E, and explored the molecular mechanism of *BmERR* response to 20E.

## Materials and Methods

### Insect

The silkworm strain of *dazao* was provided by the silkworm gene bank in the Southwest University, China.^1^ The silkworms were fed with mulberry leaves at 25°C and grown with a photoperiod of 12 h light/12 h dark and 55 ± 5% relative humidity.

### DNA/RNA Extraction and cDNA Synthesis

Previous studies found that the fat body of silkworms has the expression of *BmERR* (Shen et al., 2018). So, in this article, the fat body was used for the extraction of total RNA. The total RNA was extracted using the Trizol extraction kit (Invitrogen, United States), then digested carefully with DNase I (TAKARA, Japan) to avoid genomic DNA contamination. For race polymerase chain reaction (PCR), the first-stand cDNA of fat body was synthesized with the SMARTer RACE 5′/3′ kit (TAKARA, Japan) as the manufacturer instructions and then stored at −20°C. For the quantitative real time-PCR (qRT-PCR), M-MLV reverse transcriptase (Promega, United States) was used to generate BmE cells’ first-strand cDNA. Genomic DNA was extracted from the whole silkworm using the Tissue DNA kit (OMEGA, United States) as the manufacturer’s instruction.

### Quantitative Real Time-Polymerase Chain Reaction

Quantitative real time-PCR was performed to evaluate the expression levels of *BmERR* (GenBank: KT268294), *BmEcR* (GenBank: L35266), and *BmUSP* (GenBank: U06073.1) using the SYBR Premix Ex TaqTM (TAKARA Biotech, Japan) and an ABI StepOne v2.1 Sequence Detection System (Applied Biosystems, United States). The relative mRNA expression levels of target genes were calculated with the 2^–ΔΔCT^ method and the silkworm translation initiation factor 4A (*BmTIF4A*, NM_001043911.1) was used as an endogenous control. The primers used for PCR were listed in Table 1.

**TABLE 1:** Primer for this study.

Primer name	Purpose	5′-3′	Sequence	Remark
*BmERR*	qRT-PCR	F	CGCCGACCTGTACGACC	259 bp
		R	CACGCCCGACACCTGTAGAAA	
*BmTIF4A*	qRT-PCR	F	TTCGTACTGGCTCTTCTCGT	196 bp
		R	CAAAGTTGATAGCAATTCCCT	
*BmERR*	5′RACE	F	CTAATACGACTCACTATAGGGCAAGCAGTGTATCAACGCAGAGT	
		R	ACGGTCACTAAAGCATCGACG	
	RNAi	F	TAATACGACTCACTATAGGGAGACCGCGTCAAACAGGAAACGGATC	5′terminal
		R	TAATACGACTCACTATAGGGAGACCAGCACCTTGATGTCGTCGAG	T7 promoter
BmEcR	qRT-PCR	F	ACTTGGCAGTCGGATGAAG	66 bp
		R	CGTCATCTCCGTGATCTGG	
	RNAi	F	TAATACGACTCACTATAGGGAGAACGGTCCAGTTGATCGTCGAGTT	5′terminal
		R	TAATACGACTCACTATAGGGAGACAGCTTCAGCGAGACACATGTTG	T7 promoter
	Overexpression	F	AGGATTGGTGGATCCATGAGAGTCGAGAACGTGGATAACG	
		R	AGTTGTAGCGGCCGCCTATAGCACCACCGGGTTGGTG	
BmUSP	qRT-PCR	F	TCAAATAGGCAACAAACAGATAGCCGCTC	157 bp
		R	CAGGAACTCCATAGACCG	
	Overexpression	F	AGGATTGGTGGATCCATGTCGAGCGTGGCGAAG	
		R	AGTTGTAGCGGCCGCCTACATGATGTTGGTGTCGATGG	
EGFP	RNAi	F	TAATACGACTCACTATAGGGAGATGCTTCAGCCGCTACCC	5′terminal
		R	TAATACGACTCACTATAGGGAGATCCAGCAGGACCATGTGAT	T7 promoter
pGL3-*BmERRP* (complete promoter)	Vector for cell expression	F	cgagctc ATTAAGTAGCAGTAAACTGTGACC	*Sac*I
		R	ccgctcgag ACGGTCACTAAAGCATCGACG	*Xho*I
pGL3-*BmERRP* (truncated promoter)	Vector for cell expression	F	cgagctc ATTAAGTAGCAGTAAACTGTGACC	1,334 bp
		F	cgagctc CCTGATGGTACTTTAG	1,206 bp
		F	cgagctc TAGTCAACTCTTTGCCCCTG	841 bp
		F	cgagctc GAAAAATGTAATTGTGTTGCCAGG	520 bp
		F	cgagctc ATTTGATTTAAATTAATTTGAACCC	251 bp
		F	cgagctc TTTGAACCCAATGTTTTGCG	235 bp
		R	ccgctcgag TGAATTAAATTTAGAATATCAGCTAACGC	
Bio-ERRE-1	EMSA	F	ATTAAGTAGCAGTAAACTGTGACCT	3′–ends biotin
		R	AGGTCACAGTTTACTGCTACTTAAT	labeled
Bio-ERRE-1 mut		F	ATTAGACGATGACGGGTCATGACCT	
		R	AGGTCATGACCCGTCATCGTCTAAT	
Bio-ERRE-2		F	TAAAGAACCTTTATTAAAATTAAAATA	
		R	TATTTTAATTTTAATAAAGGTTCTTTA	
Bio-ERRE-3		F	GTTCCGAAATAAAATTACCTGATGGTA	
		R	TACCATCAGGTAATTTTATTTCGGAAC	
Bio-ERRE-8		F	GAGACAGCGTTAGCTGATATTCTAAAT	
		R	ATTTAGAATATCAGCTAACGCTGTCTC	
pGL3-EcRE-VgP78ML	Vector for cell expression	F	ccgctcgagATTAAGTAGCAGTAAACTGACGGTCTCGATCAGCG	*Xho* I
		R	cccaagcttTGATCTAGCTCCGCTGTC	Hind III
pGL3-EcRE-M-VgP78ML		F	ccgctcgagATTAGACGATGACGGGTCAACGGTCTCGATCAGCG	*Xho* I
		R	cccaagcttTGATCTAGCTCCGCTGTC	Hind III

*Different capital letters are the sequence of primers and the different small letters are the recognition sequence of restriction endonucleases.*

### Cloning 5′-Untranslated Region of *Bombyx mori* Estrogen-Related Receptor

To obtain the complete *BmERR* 5′-UTR sequence and the transcription start site (TSS), RACE was employed to clone the 5′-untranslated region of *BmERR*. The universal primer mix (UPM) from the 5′-Full RACE Kit (TAKARA, Japan) was used as the forward primer, and the specific reverse primer BmERR-R (Table 1) was designed based on the partial *BmERR* 5′-UTR sequence in our previous fat body transcriptome sequencing results (date not shown). The 5′-UTR was amplified by nested PCR using the synthesized cDNA as a template under the following program: 30 cycles of 98°C for 10 s, 55°C for 15 s, and 72°C for 30 s. The PCR products were cloned into the pMD19-T simple clone vector (TAKARA, Japan) and then sequenced.

### Bioinformatical Analysis

The upstream sequences of *BmERR* were obtained by 5′RACE-PCR and transcriptome sequencing of silkworm fat body. The *cis*-acting regulatory elements (CREs) in the upstream sequences of *BmERR* were predicted using JASPAR.^2^

### Vector Construction

Using the high-fidelity DNA polymerase (TransGen Biotech, China), different lengths of *BmERR* promoter fragments were cloned from the silkworm genomic DNA with different primers (shown in Table 1) under the following program: 95°C for 5 min; 35 cycles of 95°C for 30 s, 56°C for 30 s, and 72°C for 2 min; and 72°C for 10 min, and then inserted into the firefly luciferase reporter vector pGL3-Basic (Promega, United States) between *Sac*I and *Xho*I (TAKARA, Japan) restriction sites.

The psl 1180-HR3-A4-DsRed-SV40 (Liu et al., 2019) vector was stored in our laboratory and used for overexpression in *B. mori* cells. Using the ligation free cloning kit (abm, China), the open reading frame (ORF) of *BmEcR* and *BmUSP* were amplified from the cDNA and cloned into psl 1180-HR3-A4 SV40 vector restricted with *Bam*HI and *Not*I in the manner of homologous recombination. The primers were given in Table 1.

### Cell Transfection, Hormone Treatment, and Luciferase Assay

The luminescent reporter assay was performed according to the manufacturer’s instruction. The *B. mori* embryonic cell line (BmE) was cultured in a 24-wells plate at 27°C in Grace (Gibco, United States) insect cell culture medium supplemented with 10% fetal bovine serum (FBS) (Gibco, United States). After 12 h, every well was transfected with a mixture of 1 μg recombinant plasmid, 0.1 μg internal control plasmid pRL-78ML (Liu et al., 2018; Shen et al., 2018), and 3 μL of Lipofectamine 2000 (Invitrogen, United States) in the insect medium without FBS. After 6 h, the transfection mixture was replaced with 500 μL fresh insect medium containing 10% FBS.

Twenty-hydroxyecdysone (Sigma, United States) were dissolved in DMSO (Sigma, United States) at a stock concentration of 5 mg/mL and stored at −20°C. After 6 h of cell transfection, different concentrations of 20E were added to the 24-well cell culture plate, and the equal amount of DMSO was added as a control. The cells were harvested after 48 h of transfection and assayed with the Dual-Luciferase Reporter System (Promega, United States).

### Electrophoretic Mobility Shift Assay

Oligonucleotide sequences of four EcRE-like motifs 1/2/3/8 (EcRE-like 1/2/3/8) predicted at positions −1,325 to −1,311 (5′-AGTGCAGTAAACTGT-3′), −1,258 to −1,244 (5′-ACCTTTATTAAATT-3′), −1,199 to −1,185 (5′-TAGTGGTACTTTAGA-3′), and −28 to −14 (5′-GCGTTAGCTGATATT-3′) in the *BmERR* promoter were synthesized as probes for electrophoretic mobility shift assay (EMSA) (Sangon Biotech, China). The single-stranded sequences were labeled with biotin at the 3′-end and annealed to produce a double-stranded probe. To evaluate interactions between the regulatory elements and prokaryotic expressed proteins BmEcR and BmUSP (Shen et al., 2018), EMSA was performed as previously described using a Chemiluminescent EMSA Kit (Beyotime, China) as the manufacturer’s instructions. After incubation at 25°C for 25 min, the reaction mixtures were loaded to 5% native polyacrylamide gels and electrophoresis was conducted in Tris–borate—EDTA buffer (1 mM EDTA and 45 mM Tris–borate, pH 8.3). The proteins were transferred to the nylon membrane (Roche, United States). and then imaged with the enhanced chemiluminescence using a Clinx ChemiScope 3400 Mini system (Science Instruments, China) after incubation with Streptavidin-horseradish peroxidase.

### Double-Stranded RNA Interference

A double-stranded RNA interference (dsRNAi) approach was performed to evaluate the relationships among *BmERR, BmECR*, and *BmUSP*. The 584- and 504-bp fragments of *BmERR* and *BmECR* were, respectively, selected to synthesize double-stranded RNA (dsRNA) (Jin et al., 2020; Long et al., 2020). The fragments containing the bacteriophage T7 promoter sequence were obtained through PCR and then cloned into the pMD19-T simple vector (TAKARA, Japan). The dsRNA was generated using the T7 RiboMAX Express RNAi System (Promega, United States). A fragment of enhanced green fluorescence protein (EGFP) (458 bp) was used as a negative control. After 20 min of incubation, the mixture (5 μg dsRNA and 10 μL Lipofectamine 2000) (Invitrogen, United States) was added into the BmE cells. The primer sequences were shown in Table 1.

### Statistical Analysis

The results are presented as the mean ± SD from three independent experiments. Statistical analyses were performed using Microsoft excel (Microsoft, United States). Differences between groups were analyzed with Student’s *t*-tests, and *P < 0.05, ^**^P < 0.01, and ^***^P < 0.001 were accepted as statistically significant.

## Results

### Cloning and Activity Analysis of the *Bombyx mori* Estrogen-Related Receptor Promoter

**FIGURE 1:**
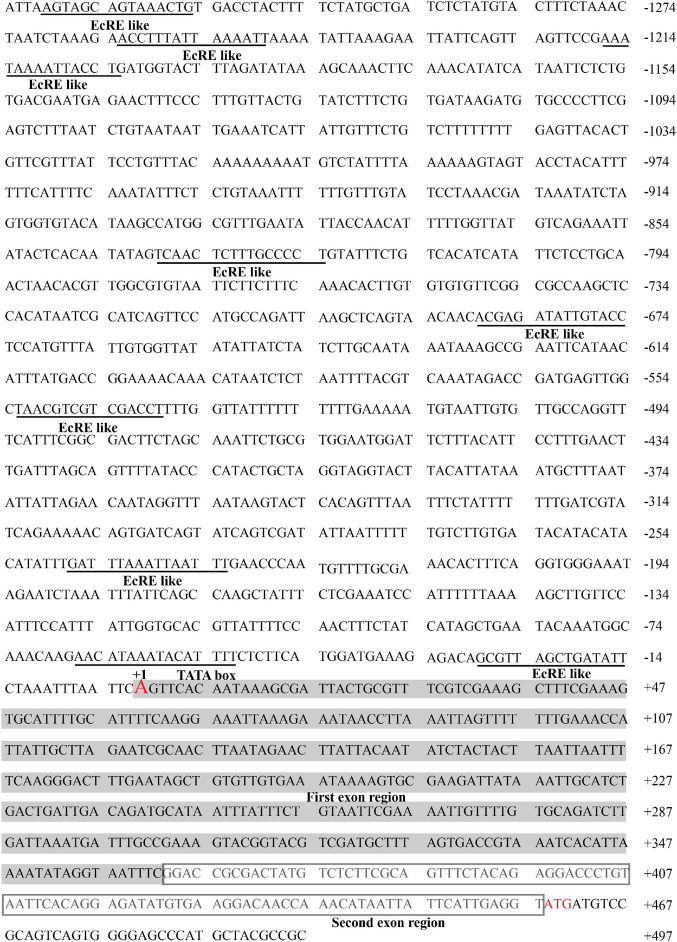
Sequence analysis of the 5′-flanking region of *Bombyx mori ERR* (*BmERR*). The nucleotides are numbered relative to the translation start site indicated by + 1, with upstream sequences preceded by “–.” The transcription start site (TSS) and translation start site are indicated in red. The putative transcription factor (TF)-binding sites are labeled. The light gray area represents the first exon sequence and the dark box represents the second exon sequence. Eight putative EcRE motif are predicted on the *BmERR* promoter.

To identify the key sequence of *BmERR* promoter, we analyzed the upstream region of *BmERR* from the silkworm genome database.^3^ The TSS and complete (5′-UTR) sequence were successfully identified through 5′-RACE PCR (Figure 1). The TSS was located 458 bp upstream of the translation initiation site and the 5′-UTR sequence was transcribed and spliced by two exon regions. Using the online website^4^, we predicted a typical TATA BOX in the region from −64 to −57 bp and eight putative ecdysone response elements (EcREs, shown in the Table 2) in −1,333 bp upstream of the TSS.

**TABLE 2:** The predicted ecdysone response elements (EcREs) on the *Bombyx mori ERR (BmERR)* promoter.

Name	Start	End	Strand	Sequence
EcRE-like 1	−1,329	−1,315	(−)	AGTAGCAGTAAACTG
EcRE-like 2	−1,262	−1,248	(+)	ACCTTTATTAAAATT
EcRE-like 3	−1,216	−1,202	(−)	AAATAAAATTACCTG
EcRE-like 4	−836	−822	(+)	CAACTCTTTGCCCCT
EcRE-like 5	−686	−672	(+)	CGAGATATTGTACCT
EcRE-like 6	−550	−536	(+)	AACGTCGTCGACCTT
EcRE-like 7	−246	−232	(−)	GATTTAAATTAATTT
EcRE-like 8	−28	−14	(+)	GCGTTAGCTGATATT

*Different capital letters are the nucleotide sequence of predicted EcREs.*

**FIGURE 2:**
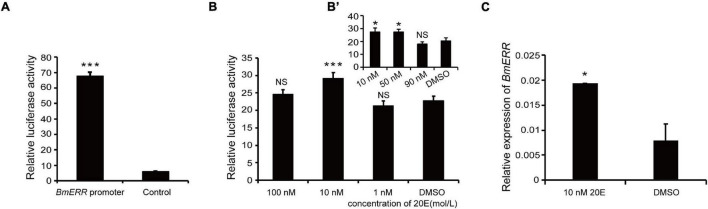
Effect of 20-hydroxyecdysone (20E) on *Bombyx mori ERR* (*BmERR*) promoter activity. **(A)** Comparison of transcriptional activity between the *BmERR* promoter and control pGL3-basic plasmids. The luciferase activity was corrected for transfection efficiency with the *Renilla* luciferase pRL-78ML plasmid and is presented as ratios to the activity of the *BmERR* promoter. **(B,B’)** Changes of *BmERR* promoter activity with different concentrations of 20E. **(C)** Quantitative real time-PCR (qRT-PCR) detection of *BmERR* expression in BmE cells treated with 10 nM 20E. Dimethyl sulfoxide was used as the control treatment. *P < 0.05, ***P < 0.001, and *t*-test.

To verify the transcriptional activity of the *BmERR* promoter, the 1,333 bp promoter fragment was amplified with a specific primer (shown in the Table 1) from silkworm genomic DNA. A cell expression vector based on the dual luciferase reporter system was constructed and then constructed vector was transfected into BmE cells. The promoter activity was measured post 48 h of transfection using a dual luciferase reporter system. Compared to the control, the *BmERR* promoter showed higher transcriptional activity (Figure 2A).

### Effect of 20-Hydroxyecdysone on *Bombyx mori* Estrogen-Related Receptor Promoter Activity

To validate the effect of 20E on BmERR promoter activity, BmE cells were transfected with BmERR promoter in the presence of different concentrations (100, 10, and 1 nM) 20E. After 48 h, *BmERR* promoter activity was up-regulated only by 10 nM (Figure 2B) and 50 nM 20E (Figure 2B’). In addition, exposure of BmE cells to 10 nM 20E for 48 h resulted in a similar effect that the transcriptional level of *BmERR* significantly increased (Figure 2C).

### The *Bombyx mori* Estrogen-Related Receptor Promoter Responds to 20-Hydroxyecdysone *via* Ecdysone Response Element

**FIGURE 3:**
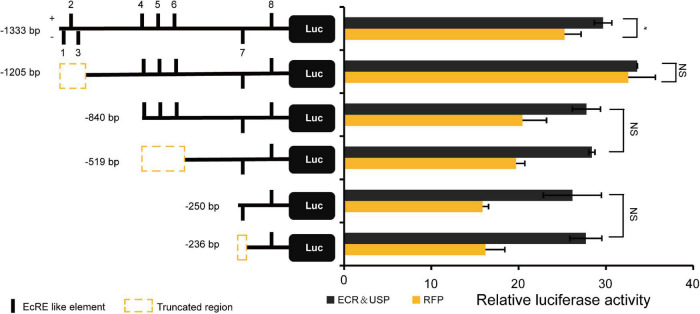
Regulation of EcRE on the *Bombyx mori ERR* (*BmERR*) promoter. Comparison of the activity of different truncated forms of *BmERR* promoter with the overexpression of *BmEcR* and *BmUSP* and *RFP* through the dual luciferase reporter system. 1–8, EcRE like 1–8, respectively. EcR and USP, the overexpression of BmEcR and BmUSP protein. *RFP*, the overexpression of red fluorescent protein (RFP). *P < 0.05. NS, not significant; *t*-test.

Eight EcRE-like elements were predicted at −1,329 to −1,315 bp, −1,262 to −1,248 bp, −1,216 to −1,202 bp, −836 to −822 bp, −686 to −672 bp, −550 to −536 bp, −250 to −236 bp, and −28 to −14 bp on the *BmERR* promoter, and designated EcRE-like 1–8, respectively. We constructed six vectors for cell transfection with different lengths of *BmERR* promoter fragments as the position of these elements on the promoter. The effects of EcRE-like elements on promoter activity were evaluated by overexpression of *BmEcR* and *BmUSP* and *Red fluorescent protein* (RFP) was transfected as a control in BmE cells. The luciferase activity assay showed that *BmERR* promoter activity was significantly increased compared with that of the control when BmEcR and BmUSP proteins were overexpressed. However, compared with the control, when the promoter fragments containing EcRE-like 1/2/3 were removed, there was no significant change in *BmERR* promoter activity, indicating that these elements may play a positive regulatory role. After the promoter fragment containing the EcRE-like elements 4/5/6/7 were truncated, respectively, the promoter activity did not show significant difference under the case of overexpression of BmEcR and BmUSP. But compared with the control, the promoter still showed obviously higher transcriptional activity (Figure 3). These findings suggested that EcRE-like elements 1–3 and 8 are likely involved in the regulation of *BmERR* promoter activity mediated by EcR/USP.

**FIGURE 4:**
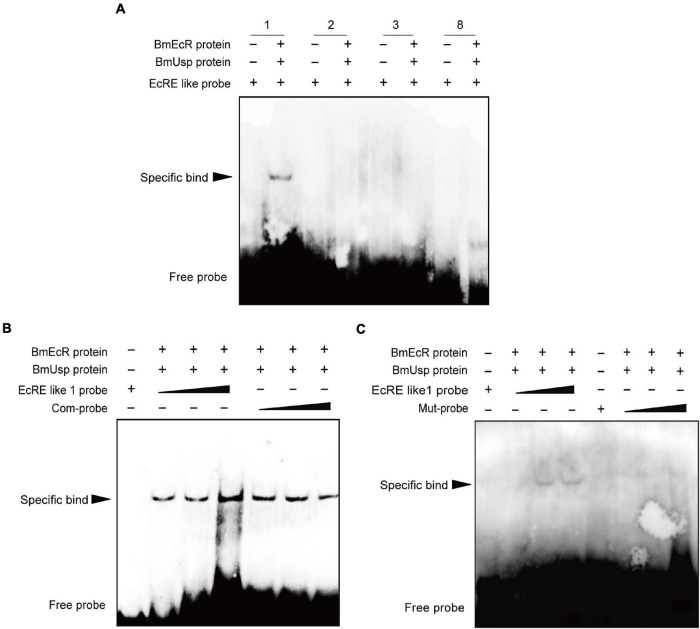
Identification of EcRE on the *Bombyx mori ERR* (*BmERR*) promoter. **(A–C)** DNA-binding activity of EcRE-like 1/2/3/8 probes with purified BmEcR and BmUSP protein verified by electrophoretic mobility shift assay (EMSA). 1/2/3/8, biotin-labeled EcRE-like 1/2/3/8 probe. Com-probe is the biotin-unlabeled competition probe which is of the same sequence as EcRE-like 1. Mut-probe is the mutated biotin-labeled EcRE-like 1 probe. The final concentration of EcRE probe was 5 μM, the concentration of BmEcR protein is 0.3 μg/μL and the concentration of BmUSP protein is 0.45 μg/μL. The ratio of EcRE probe to the competing probe is 1:10.

Electrophoretic mobility shift assay was performed to further identify these EcRE-like elements. Each biotin-labeled probe was incubated with BmEcR and BmUSP protein. Only an obvious band shift was evident after incubation with the EcRE-like 1 labeled probe (Figures 4A,B). The concentrations of the labeled probes were 50, 500, and 5 μM. The concentrations of the competing probes were 1, 5, and 50 μM, respectively. No band appeared when the sequence of EcRE-like 1 probe 5′- ATTAAGTAGCA-GTAAACTGTGACCT-3′ was mutated to 5′- ATTAGACGATGACGGGTCATG ACCT -3′ (Figure 4C).

**FIGURE 5:**
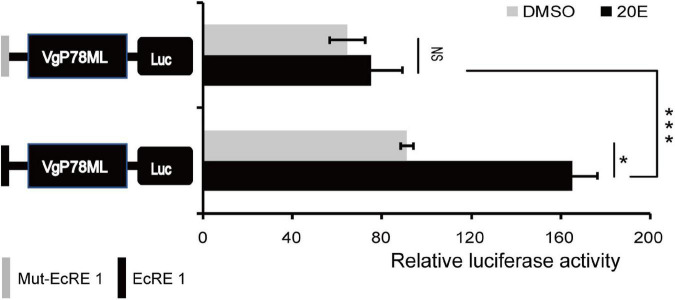
Effect of 10 nM 20-hydroxyecdysone (20E) on the EcRE-1. Luciferase activity of the basic promoters containing EcRE-1 and mut-EcRE 1 after treatment with 10 nM 20E and DMSO, respectively. Mut, mutated, NS, not significant. *P < 0.05, ***P < 0.001; *t*-test.

To further study whether the EcRE motif can respond to 20E, recombinant vectors were constructed by inserting EcRE-like 1 motif and the mutated EcRE-like 1 motif (Mut-EcRE 1) into a basal vector pGL3- VgP78ML (Liu et al., 2019), which does not respond to 20E, designated EcRE 1-P and Mut-EcRE 1-P, respectively. These two vectors were transfected into BmE cells followed by treatment with 10 nM 20E. The luciferase assay showed that only the activity of EcRE-1-P was significantly up-regulated after 20E treatment (Figure 5), suggesting that EcRE1 motif responds to 20E to up-regulate the basic promoter activity.

### Effect of Double-Stranded RNA Interference on *Bombyx mori* Estrogen-Related Receptor, *Bombyx mori* Ecdysone Receptor, and *Bombyx mori* Ultraspiracle Expression

**FIGURE 6:**
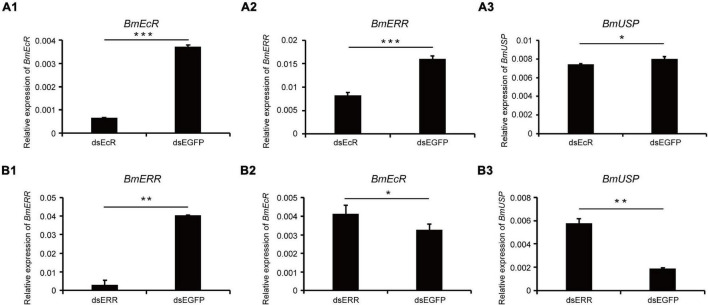
Expression levels of *Bombyx mori ERR* (*BmERR*), *BmEcR*, and *BmUSP* in BmE cells after dsRNA interference. **(A1–A3)** Expression levels of *BmERR*, *BmEcR*, and *BmUSP* after treatment with dsEcR. **(B1–B3)** Expression levels of *BmERR*, *BmEcR*, and *BmUSP* after treatment with dsERR. *P < 0.05, **P < 0.01, and ***P < 0.001; *t*-test.

In order to further explore the mechanism of 20E regulating the transcription activity of *BmERR*, the relationship between *BmERR*, *BmEcR*, and *BmUSP* was evaluated by RNA interference in BmE cells. qRT-PCR showed that the expression of *BmEcR* was significantly decreased after the dsBmEcR fragment was transfected into BmE cells (Figure 6A1), and the expression of *BmERR* and *BmUSP* were significantly reduced compared with the control (Figures 6A2,A3). RNAi of *BmERR* caused a significant reduction of *BmERR* expression (Figure 6B1). Contrast with the effects of dsBmEcR, the expression of *BmEcR* and *BmUSP* were significantly increased (Figures 6B2,B3). These results suggested a complicated network of cross-talking between BmERR, BmEcR, and BmUSP.

## Discussion

Twenty-hydroxyecdysone is an important hormone that regulates the growth, development, metabolism, and apoptosis of insects (Thummel and Chory, 2002; Yang et al., 2014). High 20E titer inhibited the expression of ERR in *Drosophila*, and the expression of genes related to carbohydrate metabolism were also significantly reduced (Kovalenko et al., 2019). Here, we cloned the nucleotide sequence of the *BmERR* promoter and confirmed that *BmERR* promoter can respond to 20E. EMSA showed BmEcR and BmUSP likely bind to the EcRE-like 1 motif on the *BmERR* promoter. After mutation of EcRE-like 1 motif, the basal promoter will not respond to 20E. This indicated that 20E up-regulated the transcriptional activity by activating the BmEcR and BmUSP complex to bind to the EcRE motif on the *BmERR* promoter.

*Bombyx mori* estrogen-related receptor and BmEcR have functional cross-talking in the BmE cells. Decreasing the expression of *BmEcR* by dsRNAi reduced the mRNA levels of both *BmERR* and *BmUSP*, whereas the expression of *BmEcR* and *BmUSP* were increased when the expression of *BmERR* was down-regulated. This is similar to the research on other insects. In the *Teleogryllus emma*, ERR and EcR both affected the development of testes. The expression of *TeEcR* and *TeERR* are regulated by each other (Jin et al., 2017). In Drosophila, EcR and ERR jointly regulated carbohydrate metabolism (Kovalenko et al., 2019). These implied that ERR may function in insects by participating in the 20E signaling pathway.

Twenty-hydroxyecdysone regulated the expression of glycolysis-related genes in the fat body of the silkworm through the ecdysone receptor EcR-USP (Tian et al., 2013; Keshan et al., 2017). In Antheraea pernyi, 20E participated in trehalose catabolism by regulating the expression of trehalase gene (Li et al., 2020). These indicated that 20E was closely related to the energy metabolism of insects. In addition, ERR was involved in carbohydrate metabolism, hypoxic metabolism and energy metabolism in Drosophila (Tennessen et al., 2011; Li et al., 2013; Kovalenko et al., 2019). Our previous research also found that BmERR regulated the expression of glycolysis-related genes to participate in the development of silkworm embryos, and affected the glucose concentration in the midgut by regulating the expression of trehalase (Long et al., 2020; Shen et al., 2021). These further implied that ERR might regulate 20E signaling by mediating nutritional metabolism, then affects insulin pathway and finally exerts its influence on 20E signaling.

So far, the research on ERR mainly focused on the function and the role in the 20E signaling pathway in insects. Previous report showed that 1 μM 20E inhibited the expression of *ERR* in Drosophila larvae, but there was no significant difference in S2 cells treated with 0.3 μM 20E (Kovalenko et al., 2019). It showed that the expression of *ERR* was very sensitive to the concentration of 20E. Our research found that 10 and 50 nM 20E could up-regulate the activity of *BmERR* promoter. Although eight EcRE motifs were predicted on the 1,333 bp *BmERR* promoter region, there was only one EcRE motif response to 20E. In addition, the 1,333 bp *BmERR* promoter region had significant transcriptional activity, but it might not contain all hormone response elements completely. It was reported that the distal sequence of the promoter also contained the motifs which respond to 20E (Nishita, 2014). These results supplied that the expression of *BmERR* was not only very sensitive to the dose of 20E, but also depended on length of the *BmERR* promoter region. In summary, our study analyzed how 20E regulate the expression of *ERR* and provided a perspective in the regulation of *ERR* expression in insects.

## Data Availability Statement

The original contributions presented in the study are included in the article/supplementary material, further inquiries can be directed to the corresponding authors.

## Author Contributions

JW and GS contributed to the conception and design of the study. JW performed the statistical analysis and wrote the first draft of the manuscript. All authors contributed to experiment, manuscript revision, read, and approved the submitted version.

## Conflict of Interest

The authors declare that the research was conducted in the absence of any commercial or financial relationships that could be construed as a potential conflict of interest.

## Publisher’s Note

All claims expressed in this article are solely those of the authors and do not necessarily represent those of their affiliated organizations, or those of the publisher, the editors and the reviewers. Any product that may be evaluated in this article, or claim that may be made by its manufacturer, is not guaranteed or endorsed by the publisher.

## Footnotes

1
http://sklsgb.swu.edu.cn/

2
http://jaspar.genereg.net/

3
https://silkdb.bioinfotoolkits.net/-main/species-info/-1

4
http://jaspardev.genereg.net/
